# Unilateral contralesional versus bilateral repetitive transcranial magnetic stimulation for patients with post stroke dysphagia: a randomized controlled study

**DOI:** 10.1007/s10072-025-08298-1

**Published:** 2025-06-23

**Authors:** Basem Hamdy Fouda, Ahmed Mostafa Kishk

**Affiliations:** Neuropsychiatry Department, Faculty of Medicine, Tanta University, Tanta, Egypt

**Keywords:** Dysphagia, Stroke, Repetitive transcranial magnetic stimulation, Swallowing rehabilitation, Neuroplasticity

## Abstract

**Background:**

Dysphagia is a frequent and challenging complication after stroke, severely impacting both recovery and quality of life. Conventional rehabilitation approaches often yield limited results, underscoring the need for novel treatments. This work compared the effectiveness of two different repetitive transcranial magnetic stimulation (rTMS) approaches in enhancing swallowing function in post-stroke dysphagia (PSD).

**Methods:**

This prospective, randomized, controlled trial was conducted with 60 patients, 18–80 years old, diagnosed with acute ischemic stroke and suffered from PSD. Participants were randomized equally to three groups: Group 1 received bilateral facilitatory rTMS targeting both hemispheres, Group 2 received unilateral facilitatory rTMS on the contralesional hemisphere, and Group 3 received sham rTMS.

**Results:**

Motor threshold (MT) scores were similar across the three groups at baseline (date 0). At follow-up, Group 1 showed significantly lower MT scores than Group 3 at dates 1 and 3 (*P* < 0.05), with no significant difference between Group 2 and Groups 1 and 3. At date 2, Group 1 had significantly lower MT scores than both Groups 2 and 3 (*P* < 0.05), while scores between Group 2 and Group 3 were comparable. MT was comparable among the groups at date 0, 1, 2, and 3. For the Modified Rankin Scale, at date 3, Groups 1 and 2 had significantly lower scores than Group 3, with no significant difference between Groups 1 and 2.

**Conclusions:**

Bilateral facilitatory rTMS is an effective and safe intervention for improving swallowing function in PSD.

## Introduction

Dysphagia plays a significant role in influencing both the recovery process and the overall quality of life of stroke survivors [1]. This swallowing disorder can lead to a range of serious health concerns, including malnutrition, dehydration, and aspiration pneumonia, which may result in prolonged hospitalization and increased healthcare burdens. The reported prevalence of dysphagia in stroke patients varies considerably depending on the diagnostic method used for detection. Specifically, screening techniques identify dysphagia in approximately 37–45% of patients, clinical assessments detect it in 51–55% of cases, and instrumental evaluations reveal a notably higher incidence, ranging from 64 to 78% [2]. While a significant proportion of patients experience some degree of natural recovery within a few weeks following the onset of dysphagia, the extent and pace of improvement can differ considerably among individuals. Unfortunately, for many stroke survivors, persistent dysphagia leads to adverse consequences such as aspiration pneumonia, nutritional deficiencies, and an increased dependence on enteral feeding [3].

Given these potential risks, a deeper understanding of the underlying mechanisms responsible for dysphagia in stroke patients is critical for developing effective therapeutic strategies. Beyond the physical complications, dysphagia can also impose substantial emotional and psychological burdens, contributing to anxiety, depression, and reduced social participation. These challenges emphasize the need for a comprehensive, multidisciplinary approach to managing PSD, incorporating both physical rehabilitation and psychological support [4].

Traditional rehabilitation approaches for managing dysphagia typically involve a combination of swallowing exercises, dietary modifications, and compensatory techniques. However, these conventional methods often yield only partial or limited improvements, as they may not sufficiently address the neurological impairments caused by a stroke [5]. With advancements in neuroscience, researchers have increasingly focused on the concept of neuroplasticity as a means to enhance recovery. One promising intervention emerging from this research is transcranial magnetic stimulation (TMS), a non-invasive neuromodulatory technique that has gained attention for its potential in improving swallowing function [6].

TMS works by modulating neural activity in key brain regions responsible for coordinating swallowing, such as the primary motor cortex (M1) and the supplementary motor area (SMA) [7]. vidence from the literature suggests that stimulating these areas with TMS can promote neural plasticity, thereby facilitating the restoration of swallowing function and improving overall recovery outcomes [8, 9].

Furthermore, studies indicate that combining TMS with structured swallowing exercises may further enhance rehabilitation by reinforcing neural connections, improving muscle coordination, and reducing the likelihood of complications such as aspiration [10]. Thus, this study aimed to evaluate and compare the effectiveness of two different repetitive transcranial magnetic stimulation (rTMS) approaches in enhancing swallowing function in individuals with PSD.

## Methods

This prospective, randomized, controlled trial was done on 60 cases, comprising both sexes, aged 18–80 years, diagnosed with acute cerebral stroke and suffering from PSD. The research was conducted from January to December 2024, after approval from the ethical committee (approval code: 36264PR923/10/24). All patients provided informed written consent.

Participants were excluded if they had hemorrhagic stroke, a disturbed consciousness, epilepsy, history of neurosurgical operations and those with metal material (plates, pacemakers) as well as patients who receive drugs (tranquilizers and or antiepileptic) and any patient with any systemic or neurological disease possibly interfering with TMS results.

All participants underwent a comprehensive clinical assessment, including both systemic and neurological examinations, which were conducted to evaluate the patient’s condition. As an additional non‑instrumental bedside measure, the Repetitive Saliva Swallowing Test (RSST) was performed scores of ≤ 2 swallows in 30 s were taken to indicate dysphagia risk.

The severity of the stroke was assessed using the modified Rankin Score [11]. For diagnosing dysphagia, the three-stage Standardized Swallowing Assessment (SSA) was utilized [12], in which failure of any item in Sect. 1 or 2, or any abnormality during the Sect. 3 water‑swallow trials, constituted a “failed” screen (i.e. dysphagia). and the severity of dysphagia was scored based on the Dysphagia Outcome and Severity Scale (DOSS) [13], a DOSS score of ≤ 5 was used to define the presence of dysphagia. Management of cerebral ischemic stroke involved brain imaging, either through computed tomography (CT) or magnetic resonance imaging (MRI), as well as routine laboratory investigations. Treatment was administered in accordance with the standardized protocol (Protocol according to AHA/ASA guidelines 2019) [14] used by the stroke unit at the Neurology Department of Tanta University Hospital.

### Randomization and blinding

To ensure the study’s integrity, a randomized allocation method was implemented using computer-generated numbers (https://www.randomizer.org/) with each participant’s code secured in opaque, sealed envelopes to maintain blinding. Participants were equally randomized into three groups in a 1:1:1 ratio. Patients were divided into three groups: Group 1 received a bilateral facilitatory protocol targeting both cerebral hemispheres; Group 2 received a unilateral facilitatory protocol on the contralesional hemisphere; and Group 3, the control group, received sham magnetic stimulation. For sham, the coil was positioned at a 90° tilt over the same hemisphere(s) as the corresponding real‑stimulation protocol (bilaterally for Group 1; unilaterally over the contralesional side for Group 2), generating the auditory click without effective cortical stimulation.

### Repetitive transcranial magnetic stimulation

rTMS was administered to all participants, who were carefully selected from a population of patients experiencing their first-ever cerebral hemispheric ischemic stroke. The total study cohort consisted of 60 individuals, with 40 patients receiving real rTMS treatment. These 40 patients were further divided into two distinct groups based on the specific stimulation protocol applied. In addition to the treatment groups, a control group comprising 20 patients was included, receiving sham TMS to serve as a comparative baseline. Patient selection followed a consecutive and alternating pattern to ensure a balanced distribution across all groups.

Each participant underwent a structured rTMS intervention schedule, receiving a total of five consecutive daily sessions per week for a duration of two weeks. Following the completion of the rTMS treatment, patients were systematically monitored at three key time points to assess both stroke severity and the degree of dysphagia, as measured by the DOSS. The initial follow-up evaluation took place immediately after the final rTMS session (Date 1), with subsequent assessments conducted at 30 days (Date 2) and 90 days (Date 3) post-treatment. These time points were chosen to provide insight into both the immediate and longer-term effects of rTMS on swallowing function and overall neurological recovery.

To determine the resting motor threshold (MT), single‑pulse TMS was delivered to the primary motor cortex, locating the FDI hotspot. For the actual rTMS intervention, the coil was then repositioned ~ 3 cm lateral and 1.5 cm anterior to the vertex—corresponding to the pharyngeal motor cortex—where MEPs in submental muscles were monitored to confirm hotspot reliability. The MT was defined as the minimum stimulus intensity required to induce a motor-evoked potential (MEP) with an amplitude of at least 50 µV in five out of ten consecutive trials. These MEP responses were collected from the afflicted upper limb’s first dorsal interosseous (FDI) muscle, which ensured measurement and analytical accuracy.

The rTMS pulses were delivered using a figure-of-eight coil connected to a Magstim Rapid2 stimulator (Magstim Co., UK). To optimize stimulation accuracy, the coil was positioned tangentially against the scalp, with its handle oriented backward and laterally at a 45-degree angle relative to the mid-sagittal plane. The precise motor hotspot was identified as the scalp location where TMS consistently elicited the highest-amplitude MEP response in the targeted muscle. Once the optimal hotspot was determined, the MT was established using a standardized incremental and decremental approach. Stimulation intensity was carefully adjusted in small steps of 1–2% of the maximum stimulator output (MSO) to precisely determine the threshold level.

The MT was systematically measured at four specific time points throughout the study: immediately after the final rTMS session (Date 0), followed by evaluations at 30 days (Date 1), 90 days (Date 2), and the final follow-up session (Date 3). These data points allowed for both intra-group (within the same group) and inter-group (between groups) comparisons to assess changes in cortical excitability over time and to determine the effectiveness of rTMS in promoting neural plasticity and functional recovery.

To ensure accurate and reliable MT assessment, all measurements were performed in a controlled, quiet environment, with patients seated comfortably in a chair. Participants were instructed to remain completely relaxed during the procedure to minimize variability in MEP responses. Furthermore, to enhance data consistency and reliability, the same investigator conducted all MT measurements throughout the study, reducing the potential for inter-observer variability.

The study’s primary outcome was to assess the safety and efficacy of TMS as an early intervention for patients with post stroke dysphagia, while secondary outcomes were to document the best stimulatory protocol with better results in term of long-term safety and efficacy.

### Statistical analysis

All statistical analyses were performed using SPSS version 27 (IBM©, Chicago, IL, USA). Data normality was evaluated using the Shapiro-Wilks test and visualized with histograms. For quantitative parametric variables, results were expressed as means ± standard deviations and analyzed using ANOVA followed by Tukey’s post hoc test. Quantitative non-parametric data were presented as medians and interquartile ranges and examined with the Kruskal-Wallis’s test and Mann-Whitney post hoc tests. Qualitative variables were reported as frequencies and percentages and analyzed using the Chi-square test. A two-tailed *P* < 0.05 was considered statistically significant.

## Results

**Fig. 1:**
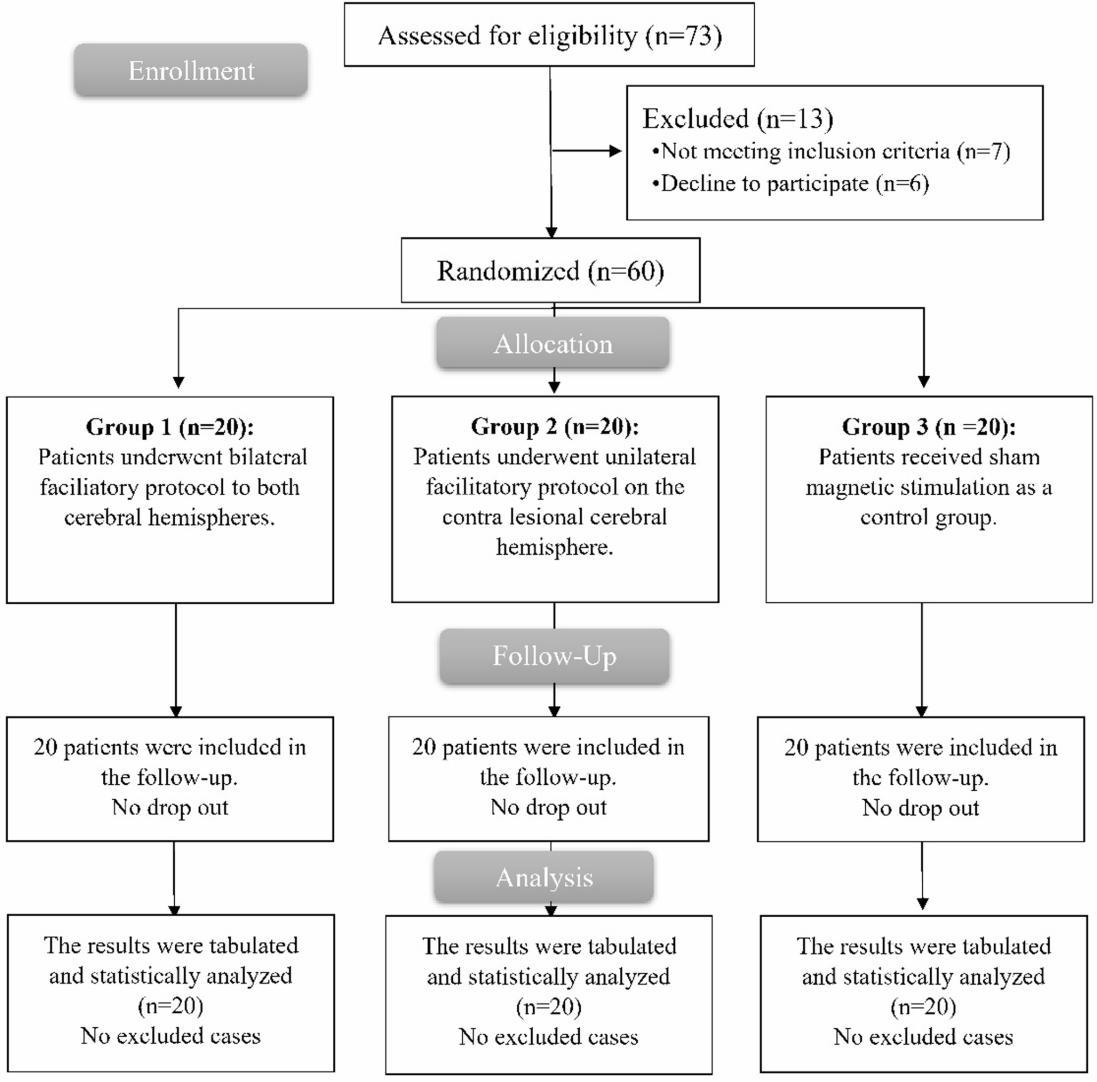
CONSORT flow chart of the enrolled patients

Figure 1 illustrates that 73 cases were enrolled, and their eligibility for participation was assessed. Seven patients did not meet the inclusion criteria and six opted out of participation resulting in a total of 60 cases that were randomized to three groups for subsequent analysis.

Table 1 indicated that all groups had comparable demographics.

**Table 1:** Demographic data of the studied groups

		Group A (*n* = 20)	Group B (*n* = 20)	Group C (*n* = 0)	*P*
Age (years)		60.6 ± 9.66	59.6 ± 9.3	60.9 ± 10.48	0.901
Sex	Male	8 (40%)	10 (50%)	11 (55%)	0.626
	Female	12 (60%)	10 (50%)	9 (45%)	
Type of stroke	Total MCA	6 (30%)	6 (30%)	6 (30%)	0.956
	partial MCA	9 (45%)	8 (40%)	10 (50%)	
	LACI	5 (25%)	6 (30%)	4 (20%)	
Work type	Manual worker	12 (60%)	11 (55%)	13 (65%)	0.811
	Hand worker	8 (40%)	9 (45%)	7 (35%)	
Side	Right	13 (65%)	9 (45%)	12 (60%)	0.413
	Left	7 (35%)	11 (55%)	8 (40%)	
Dominant hand	Right	13 (65%)	14 (70%)	12 (60%)	0.802
	Left	7 (35%)	6 (30%)	8 (40%)	

Data are presented as mean ± SD or frequency (%). BMI: Body mass index, MCA: Middle cerebral artery, LACI Lacunar cerebral infarct

Table 2 demonstrates that the motor threshold (MT) was insignificantly different at date 0, date 1, date 2 and date 3 among three groups. MT was significantly lower at date 1and date 2 than date 0 in group 1 and group 2 (*P* < 0.05) and were insignificantly different between date 1 and date 0 and was significantly lower at date 2 and date 3 than date 0 in group 3.

**Table 2:** Motor threshold of the studied groups

	Group 1 (*n* = 20)	Group 2 (*n* = 20)	Group 3 (*n* = 20)	*P*^^
**Date 0**	68.85 ± 9.24	67.4 ± 8.49	66.25 ± 8.56	0.645
**Date 1**	65.65 ± 7.98	66 ± 9.12	66.25 ± 8.56	0.976
**P^**	< 0.001*	0.035*	0.099	
**Date 2**	62.25 ± 7.52	64 ± 9.54	65 ± 9.46	0.976
**P^**	< 0.001*	< 0.001*	< 0.021*	
**Date 3**	61 ± 6.61	61.7 ± 9.71	63.5 ± 8.75	0.615
**P^**	< 0.001*	< 0.001*	< 0.001*	

Data are presented as mean ± SD, Date 0: After the last session of rTMS, Date 1: After 30 days, Date 2: After 90 days, Date 3: After the last session regarding stroke severity. P^: within-group comparison over time. P^^: Between-group comparison at each time point

**Fig. 2:**
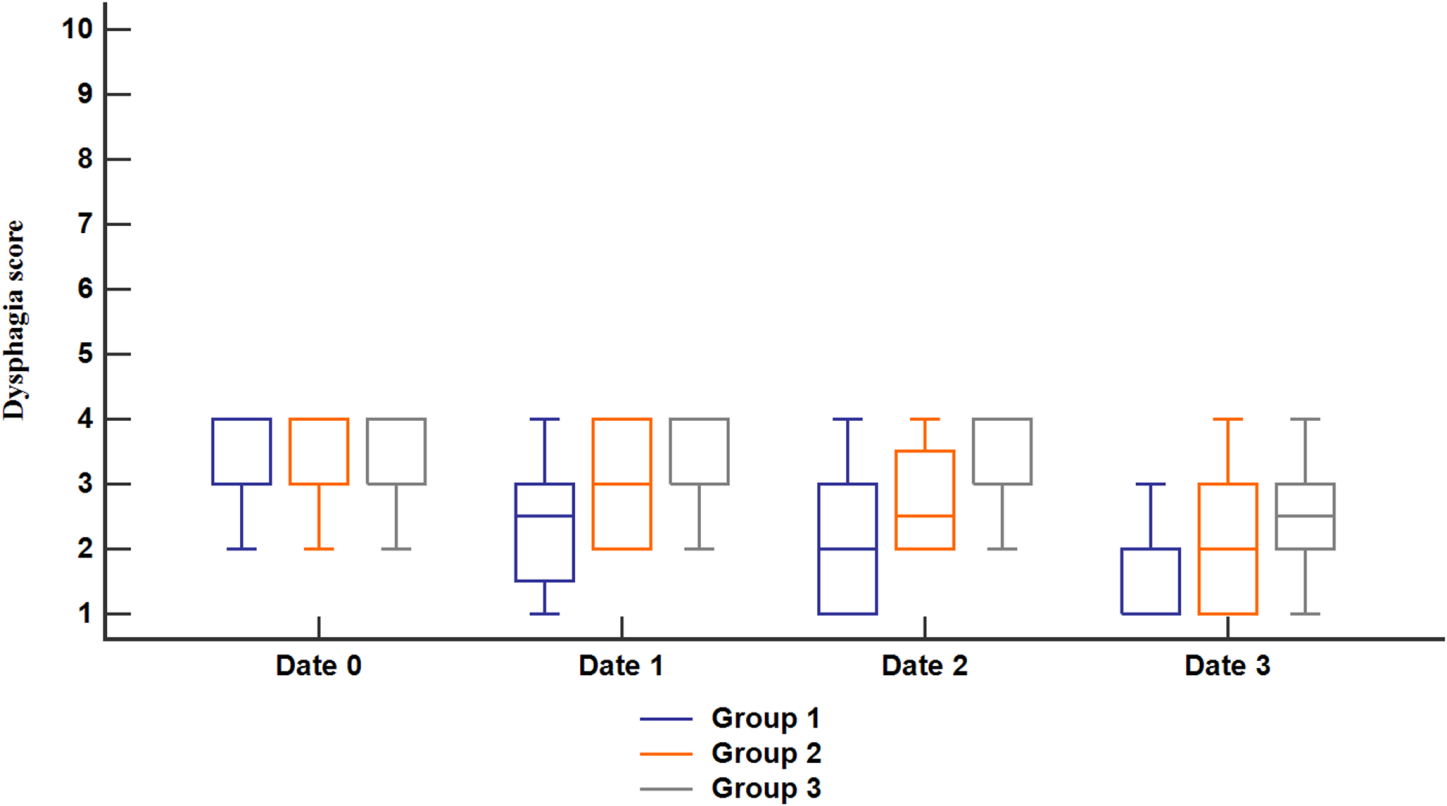
Dysphagia score of the studied groups

Figure 2 shows that Despite the observed differences in improvement, the enhancement in MRS was not significantly different between the two groups, suggesting that both the bilateral and unilateral protocols yielded similar clinical outcomes, while in account for MT specifically, in Group 1, MT decreased from 62.25 ± 7.52 at date 2 to 61 ± 6.61 at date 3, while in Group 2, MT decreased from 64 ± 9.54 to 61.7 ± 9.71. These changes indicate an improvement in excitability, with a slightly greater improvement in Group 1 compared to Group 2, suggesting that the bilateral protocol may have been more effective than the unilateral protocol.

**Fig. 3:**
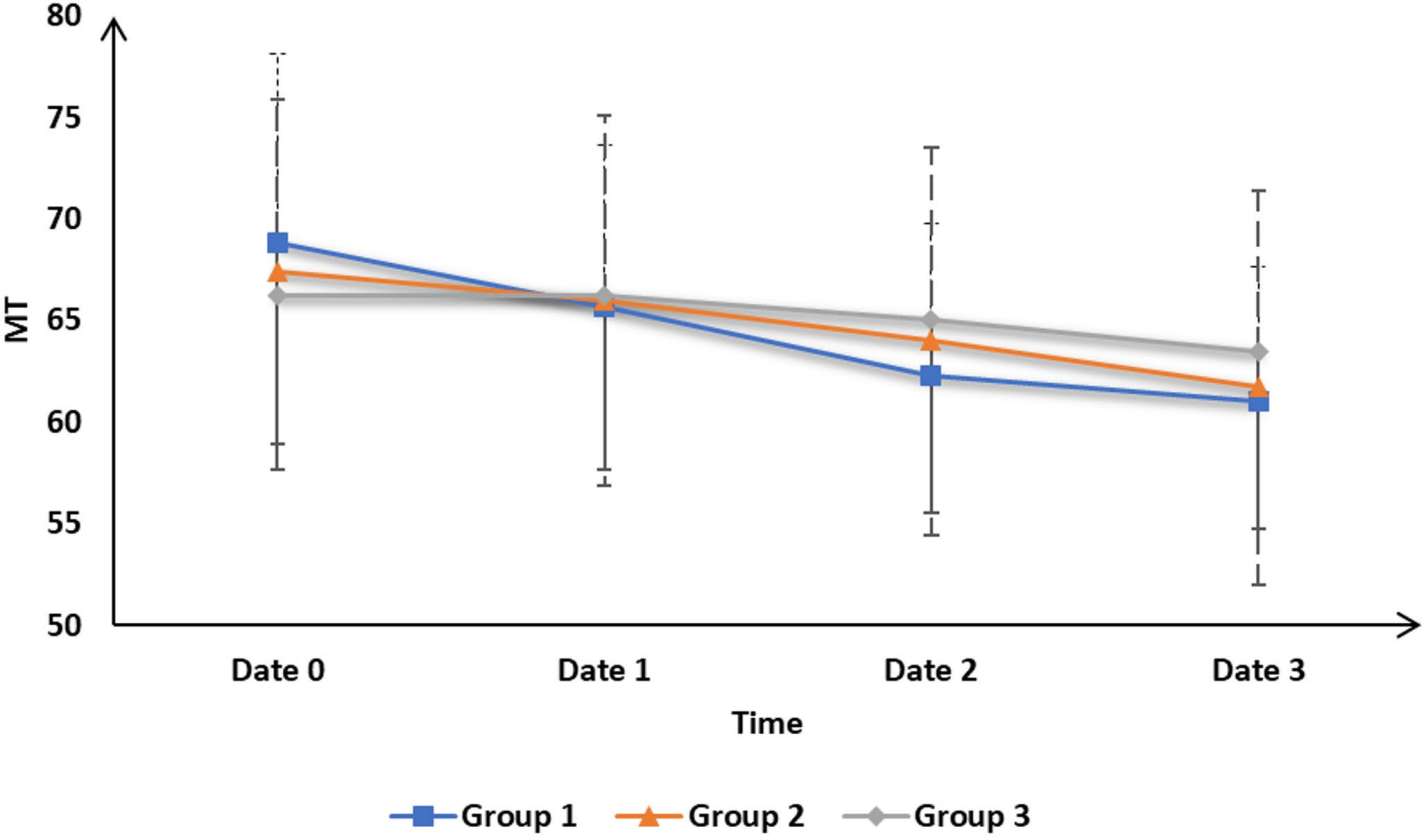
Motor threshold (MT) of the studied groups

MT decreased from 64 ± 9.54 to 61.7 ± 9.71. These changes indicate an improvement in excitability, with a slightly greater improvement in Group 1 compared to Group 2, suggesting that the bilateral protocol may have been more effective than the unilateral protocol. Figure 3.

Table 3 indicated that the modified Rankin scale (MRS) was insignificantly different at date 0 among three groups and was significantly different at date 3 among three groups (*P* < 0.05). MRS at date 3 was significantly lower in (group 1 and group 2) than group 3 (*P* < 0.05) and were insignificantly different between group 1 and group 2.

**Table 3:** Modified Rankin scale of the studied groups

	Group 1 (*n* = 20)	Group 2 (*n* = 20)	Group 3 (*n* = 20)	*P*	Post hoc
**Date 0**	3.5(3-4.25)	4(2.75-5)	4(3–5)	0.284	
**Date 3**	2.5(1.75-3)	2(1.75-4)	3.5(3–4)	0.009*	P1 = 0.874 P2 = 0.006* P3 = 0.010*

Data is presented as median (IQR). Date 0: After the last session of rTMS, Date 3: After the last session regarding stroke severity. P1: P value between group 1 and group 2, P2: P value between group 1 and group 3, P3: P value between group 2 and group 3

## Discussion

Stroke often results in varying degrees of sequelae, with PSD being a common complication, affecting up to 70% of patients [15]. PSD can cause issues such as aspiration pneumonia, malnutrition, longer hospital stays, and a significant decline in quality of life, placing a heavy burden on families and society [16]. PSD may even increase the risk of death [17].

At present, standard treatments for dysphagia involve swallowing-related muscle exercises and interventions that focus on movements and postures. While clinical efficacy has improved to some extent, these traditional swallowing exercises do not benefit all patients. Additionally, there is a lack of sufficient clinical evidence to support their effectiveness [18]. Rehabilitation plays a crucial role in enhancing recovery by integrating diverse therapeutic strategies, including neuromuscular re-education, sensory stimulation, and functional training, which aim to improve the coordination and strength of the swallowing muscles [19]. This comprehensive approach seeks to address the underlying neurological deficits, offering a more individualized and holistic treatment plan for patients with dysphagia [20].

Our choice to target the pharyngeal motor cortex (rather than the FDI hand area) aligns with prior dysphagia rTMS studies [21–23], which demonstrate that stimulation of the pharyngeal representation yields more direct modulation of swallowing musculature and functional recovery.

We noted that MT score showed no significant differences at date 0 among the three groups but was significantly lower at dates 1 and 3 in the bilateral protocol group than in the sham group and was insignificantly different between the unilateral group and both the bilateral protocol and sham groups. At date 2, the MT score was significantly lower in the bilateral protocol group than in the unilateral and sham groups, with no significant difference between the unilateral and sham groups. MT showed no significant differences at dates 0, 1, 2, and 3 among the three groups. MT in groups 1 and 2 was significantly lower at (date 1, date 2 and date 3) than date 0 and in group 3 was significantly lower at (dates 2 and 3) than date 0 while was insignificant between dates 0 and 1. MRS showed no significant differences at date 0 but was significantly different at date 3, being significantly lower in the bilateral protocol and unilateral groups than in the sham group and showing no significant difference between the bilateral protocol and unilateral groups.

In line with our results, Liu et al. [24] noticed that rTMS significantly improved swallowing function in patients with PSD, especially when combined with traditional swallowing exercises, reinforcing the effectiveness of rTMS in dysphagia recovery. However, they observed no significant differences based on the stimulation site (unilateral vs. bilateral cerebellum) or stimulation mode (rTMS vs. intermittent theta-burst stimulation). This disagreement may stem from the different brain regions targeted, as they focused on the cerebellum, which may respond differently to stimulation compared to the cerebral hemispheres targeted in our study.

In agreement with our results, Wu et al. [25] found that various rTMS protocols, particularly high-frequency (HF) stimulation applied to the ipsilesional and bilateral hemispheres, significantly improved swallowing function in patients with PSD. Their meta-analysis showed that HF/bilateral hemisphere (bi-hemi) rTMS had one of the most substantial effects. However, they found that rTMS failed to improve swallowing function in chronic stage patients. This disagreement could arise from the broader range of stroke stages analyzed by Wu et al., suggesting that the efficacy of rTMS may vary depending on the timing of the intervention post-stroke.

Additionally, Fan et al. [26] demonstrated that rTMS is effective in improving motor functions in stroke patients. While Fan et al. focused on lower limb motor function, their findings showed that HF rTMS was particularly effective in improving outcomes. However, they found that low frequency rTMS was also effective in certain contexts, particularly for balance and walking speed. This disagreement might be due to the different motor functions targeted.

Moreover, Engelhardt et al. [27] demonstrated that low frequency rTMS could facilitate recovery in post-stroke patients, particularly improving hand motor function in the short term. rTMS improved motor function one month postoperatively. However, they did not observe long-term improvements or significant differences between the rTMS and sham groups in the primary outcome (Fugl Meyer score) after three months. This disagreement may arise from differences in the intervention protocol, as they used low frequency rTMS targeting the motor cortex for upper extremity function.

Also, Park et al. [23] noted that bilateral stimulation led to significantly greater improvements in swallowing function compared to unilateral or sham stimulation protocols. However, they noted that the improvements in swallowing function were consistent across all time points assessed (T1 and T2). The cause of this disagreement may be due to the different assessment tools used as they employed comprehensive scales such as the clinical dysphagia scale, penetration aspiration scale, and videofluoroscopic dysphagia scale, which could detect subtler changes in swallowing function.

Also, Momosaki et al. [28] demonstrated that the combination of bilateral cerebral rTMS and intensive swallowing rehabilitation led to improvements in swallowing function in patients with chronic post-stroke dysphagia.

Similarly, a meta-analysis by Pisegna and colleagues [29] reviewed seven randomized controlled trials and reported that non-invasive brain stimulation facilitated recovery in post-stroke dysphagia, with a moderate and significant pooled effect size. The study also noted that both tDCS and rTMS showed similar pooled effect sizes on swallowing outcomes, but emphasized the need for further research to determine the most effective stimulation parameters and patient characteristics that may influence treatment outcomes.​.

Also, a meta-analysis by Wang and colleagues [30] included 27 randomized controlled trials with 914 stroke cases and found that non-invasive neurostimulation therapies had a significant positive impact on swallowing function in contrast with the control groups. Subgroup analyses suggested that rTMS appeared to perform better, stimulation applied in the acute phase may be more effective, patients with brainstem injuries seemed to achieve better outcomes, and the cerebral infarction group had better outcomes than the mixed group of cerebral infarction and hemorrhage.

Limitations include the exclusion of cases with certain conditions, as hemorrhagic stroke and epilepsy, which may limit generalizability. The sample size of 60 patients was relatively small, and the study focused only on acute ischemic stroke. The follow-up period of up to 90 days may not capture long-term effects, and while significant improvements in dysphagia scores were observed, no clear impact on stroke severity or motor function was noted at certain time points. Further investigations should include larger, more diverse populations, longer follow-up periods, and standardized protocols to enhance the reliability and generalizability of results. Exploring different rTMS protocols and their combination with other rehabilitation strategies could improve treatment outcomes for PSD.

## Conclusions

rTMS is an effective and safe approach for improving swallowing function in post-stroke dysphagia. Among the tested protocols, bilateral facilitatory rTMS appears to offer the most significant and sustained improvements in swallowing function, making it a promising therapeutic approach for stroke rehabilitation.

## Data Availability

Data is available on reasonable requests from corresponding author.
